# Exploring the resistance mechanism of triple-negative breast cancer to paclitaxel through the scRNA-seq analysis

**DOI:** 10.1371/journal.pone.0297260

**Published:** 2024-01-16

**Authors:** Wei Gao, Linlin Sun, Jinwei Gai, Yinan Cao, Shuqun Zhang

**Affiliations:** 1 Department of Oncology, The Second Affiliated Hospital of Xi’an Jiaotong University, Xi’an, China; 2 Day Surgery Center, Dalian Municipal Central Hospital, Dalian, China; 3 Graduate School of Dalian Medical University, Dalian, China; Affiliated Hospital of Nantong University, CHINA

## Abstract

**Background:**

The triple negative breast cancer (TNBC) is the most malignant subtype of breast cancer with high aggressiveness. Although paclitaxel-based chemotherapy scenario present the mainstay in TNBC treatment, paclitaxel resistance is still a striking obstacle for cancer cure. So it is imperative to probe new therapeutic targets through illustrating the mechanisms underlying paclitaxel chemoresistance.

**Methods:**

The Single cell RNA sequencing (scRNA-seq) data of TNBC cells treated with paclitaxel at different points were downloaded from the Gene Expression Omnibus (GEO) database. The Seurat R package was used to filter and integrate the scRNA-seq expression matrix. Cells were further clustered by the FindClusters function, and the gene marker of each subset was defined by FindAllMarkers function. Then, the hallmark score of each cell was calculated by AUCell R package, the biological function of the highly expressed interest genes was analyzed by the DAVID database. Subsequently, we performed pseudotime analysis to explore the change patterns of drug resistance genes and SCENIC analysis to identify the key transcription factors (TFs). Finally, the inhibitors of which were also analyzed by the CTD database.

**Results:**

We finally obtained 6 cell subsets from 2798 cells, which were marked as AKR1C3+, WNT7A+, FAM72B+, RERG+, IDO1+ and HEY1+HCC1143 cell subsets, among which the AKR1C3+, IDO1+ and HEY1+ cell subsets proportions increased with increasing treatment time, and then were regarded as paclitaxel resistance subsets. Hallmark score and pseudotime analysis showed that these paclitaxel resistance subsets were associated with the inflammatory response, virus and interferon response activation. In addition, the gene regulatory networks (GRNs) indicated that 3 key TFs (STAT1, CEBPB and IRF7) played vital role in promoting resistance development, and five common inhibitors targeted these TFs as potential combination therapies of paclitaxel were identified.

**Conclusion:**

In this study, we identified 3 paclitaxel resistance relevant IFs and their inhibitors, which offers essential molecular basis for paclitaxel resistance and beneficial guidance for the combination of paclitaxel in clinical TNBC therapy.

## Introduction

Breast cancer is regarded as the most common malignancy diagnosed in women worldwide [1, 2], accounting for approximately 25% of all cancer cases [3]. Immunohistochemical analysis defined five major intrinsic or molecular subtypes of breast cancer based on the expression of estrogen and progesterone receptors (ER/PR) status [4], including the Luminal A (40%), Luminal B (20%), HER2-enriched (10–15%), Normal-like (2–8%) and Triple Negative (15–20%) [2, 5]. Among which, the triple negative breast cancer (TNBC) was characterized by the highest mortality and proliferative rate [6], higher early recurrences rate, distant metastases and poor outcomes [7], accompanied with lacked expressions of ER, PR and human epidermal growth factor receptor-2 (HER2) [8]. TNBC posed a greatly threat to women’s health due to the enormous heterogeneity and the absence of available molecular targets [9]. Due to this heterogeneity, large tumors may contain multiple cells with different molecular characteristics and displaying different sensitivity to treatment [10], which has been demonstrated to be the main reason for drug resistance in breast cancer therapy [11]. Clinically, TNBC tumor presents most commonly biological aggressive ductal carcinoma [12] and tend to be larger size, higher grade at diagnosis and involves lymph node [13]. Although the TNBC with aggressive feature, about 20% patients exhibited a pathologic complete response (pCR) after pre-operative chemotherapy [14]. However, TNBC patients without pCR suffering from early recurrence and metastatic death were several times of these non-TNBC patients [15]. Overall, TNBC patients had better PCR rate and a distinctly inferior overall survival compared with these non-TNBC patients, termed as “triple negative paradox” [15], which could be due to the majority TNBC patients become resistant during treatment. Thus, it is crucial to discover novel molecular targets and develop new therapeutic strategies by enlightening the mechanisms of chemoresistance.

Chemotherapy remains currently the chief systemic treatment option for TNBC patients [16, 17], under the fact that many TNBC patients miss the surgical window at the time of late diagnosis [18] as well as display resistance to immunotherapy with no appropriate responsive predictor [19]. However, chemotherapy resistance also represents a tremendous hurdle for successful cancer cure [20], especially in the metastatic setting, which is responsible for 90% therapy failure [21]. The intrinsic drug resistance usually exists prior to drug application, the sensitive cells were selectively eliminated, promoting the growth and proliferation of resistant cells [22]. However, the acquired resistance referred to that a subset of cells occurred the genetic or epigenetic events of no response to drug exposure, leading to their survival and expansion [23]. For instance, the T790M acquired mutations in Epidermal Growth Factor Receptor (EGFR) were associated with resistance to the tyrosine kinase inhibitors inhibitor (afatinib, erlotinib and gefitinib) in lung cancer [24]. In another way, the non-genomic “adaptive resistance” is usually associated with tumor relapse and involved the rewiring of the signaling or transcriptional networks to escape drugs inhibition [25]. The signaling rewiring of adaptive resistance was caused by the perturbation of cross-talk and feedback regulatory loops, eventually leading to bypass the drug inhibition and rapidly induce chemotherapy resistance [26].

The diverse resistance mechanisms to targeted therapies robustly indicated that monotherpeutic agents may not have good effect, while the combination therapies of two or more drugs is promising in improving the effectiveness of treatment and prevent the drug resistance [27]. The progress in seeking specific drugs for TNBC is slow, and paclitaxel-based therapeutic schedule is still the first-line for TNBC treatment [28, 29]. Paclitaxel is a microtubule stabilizer that inhibits cell mitosis [30]. However, a significant number of patients develop resistance after a period of paclitaxel treatment [31] because of aforemented resistance mechanisms. For example, PPP1R14B could facilitate paclitaxel resistance in TNBC patients [32]. Therefore, exploring novel therapeutic targets that drive progression and paclitaxel chemoresistance and combination of paclitaxel with target inhibitors have potential clinical application value.

In this study, we conducted a comprehensive analysis of scRNA-seq data of TNBC cells treated with paclitaxel at different points (24 and 72h) to explore the potential resistance mechanisms to paclitaxel. Firstly, the paclitaxel resistance subsets were identified. Secondly, pathway analysis and pseudotime analysis were conducted on these paclitaxel resistance subsets. Then, the genes regulatory networks (GRNs) analysis was performed to identify crucial regulons that promoted paclitaxel resistance. Finally, five drugs were identified via Comparative Toxicogenomics Database (CTD) and could be used as combination therapies of paclitaxel to improve the survival outcome of patients with TNBC.

## Material and methods

### Data acquisition

The dataset of GSE139129 was downloaded from the Gene Expression Omnibus database (GEO, https://www.ncbi.nlm.nih.gov/gds/) [33, 34], including the TNBC cell lines (HCC1143) treated with paclitaxel for 24 and 72 hours respectively were set as experimental groups. Cells treated with dimethyl sulfoxide (DMSO) for 24 and 72 hours respectively were set as control group. Then the samples were performed the single-cell RNA-sequencing (scRNA-seq) using the Illumina NextSeq 500. The informed consent was not required because this article does not contain any studies with human participants. And all data from publicly available databases.

### Data preprocessing

The Seurat R package was used to read the scRNA-seq expression matrix [35], removing cells with a mitochondrial ratio > = 10%. SCTransform function was used to normalize the data [35], the harmony R package was used to remove batch effects between samples [36] after principal component analysis (PCA) dimensionality reduction. Then the top 20 principal components were used for Uniform Manifold Approximation and Projection for Dimension Reduction (UMAP) [37], the FindNeighbors and FindClusters function (resulotion = 0.04) was further performed for the unsupervised clustering [38].

### Identification of marker genes among cell subsets

To explore the heterogeneity of each cell subsets, the FindAllMarkers function was used to calculate the differentially expressed genes (DEGs) among cell subsets (setting min.pct = 0.25, only.pos = T and logfc.threshold = 0.25) [39], and annotated the gene markers of each subset via CellMarker databse based on the specially highly expressed genes.

### Gene function enrichment analysis

We uploaded these highly expressed differential genes to the Database for Annotation, Visualization and Integrated Discovery (DAVID, https://david.ncifcrf.gov/) website [40] and explored the biological function of these cell subsets.

### Hallmark enrichment score

We downloaded the hallmark gene set“h.all.v2023.1.Hs.symbols.gmt” from the Molecular Signatures Database (MSigDB) database [41], and calculated the hallmark enrichment score of each cell through the AUCell R package [42].

### Pseudotime analysis

We performed the monocle2 R package to read the counts expression matrix, and incorporated the cell phenotypic information [43]. Then the newCellDataSet function was used to construct the CellDataSet (cds) object, which includes the expression matrix, phenoData (cell phenotype) and featureData (gene annotation), the genes expressed in fewer than 10 cells were removed [43]. Subsequently, the FindAllmarkers function was used to identify the DEGs between the Control (24h), Treated (24h) and Treated (72h) groups (filter criteria: log2FC>0.25 and p_adj<0.01) [39], these DEGs were incorporated into the cds object through the setOrderingFilter function for trajectory construction [43]. The reduceDimension function was used for the dimensionality reduction of cds object (setting max_components = 2 and method = “DDRTree”), the orderCells function was used to complete ordering the cells and trajectory construction in pseudotime (setting Control(24h) as the trajectory start point) [43]. Lastly, we used the differentialGeneTest function to find the differential genes that change as a function of pseudotime (setting fullModelFormulaStr = “~sm.ns(Pseudotime)” and qval <0.01) [44], the plot_pseudotime_heatmap function was used to visualize these pseudotime-dependent genes, in which the interest genes were further used for scatter plot visualization through the plot_genes_branched_pseudotime function [44].

### SCENIC analysis

The cellular heterogeneity is caused by the specific transcriptional state, which is determined and maintained by transcription factors (TFs)-dominated gene regulatory networks (GRNs) [45]. Therefore, analyzing of single-cell GRNs is helpful to understand the biological significance behind cell heterogeneity. The Single-cell regulatory network inference and clustering (SCENIC) analysis is an GRNs algorithm, which introduced the motif sequence of transcriptional factor and co-expression analysis to establish the GRNs model [46]. The GENIE3 R package was used to screen genes co-expressed with TFs [47], RcisTarget R package was used for TFs motif analysis to identify the regulons (TF and target genes pair) [48], the top5perTarget was used to construct the TFs regulatory network and the AUCell function was used to assess the regulons activity score [49], which reflected the intensity of TFs regulation on its target genes.

### Identification of transcription factor inhibitor

Comparative Toxicogenomics Database (CTD, http://ctdbase.org/) is a useful tool of exploring disease-drugs-genes relationship [50]. We searched for and downloaded compounds that targeted paclitaxel resistance-related TFs, and identified the common inhibitors of these TFs.

### Statistical analysis

All statistical analysis and visualization were completed by using R software (version 4.3.1). The Pearson method was used to perform the correlation analysis between the hallmark score and pserudotime. Sangerbox (http://sangerbox.com/home.html) also provided some necessary auxiliary analyses in our study. For statistical data, a p value < 0.05 was considered as statistically significant.

## Results

### Single cell profile and paclitaxel-resistant subsets analysis

**Fig 1:**
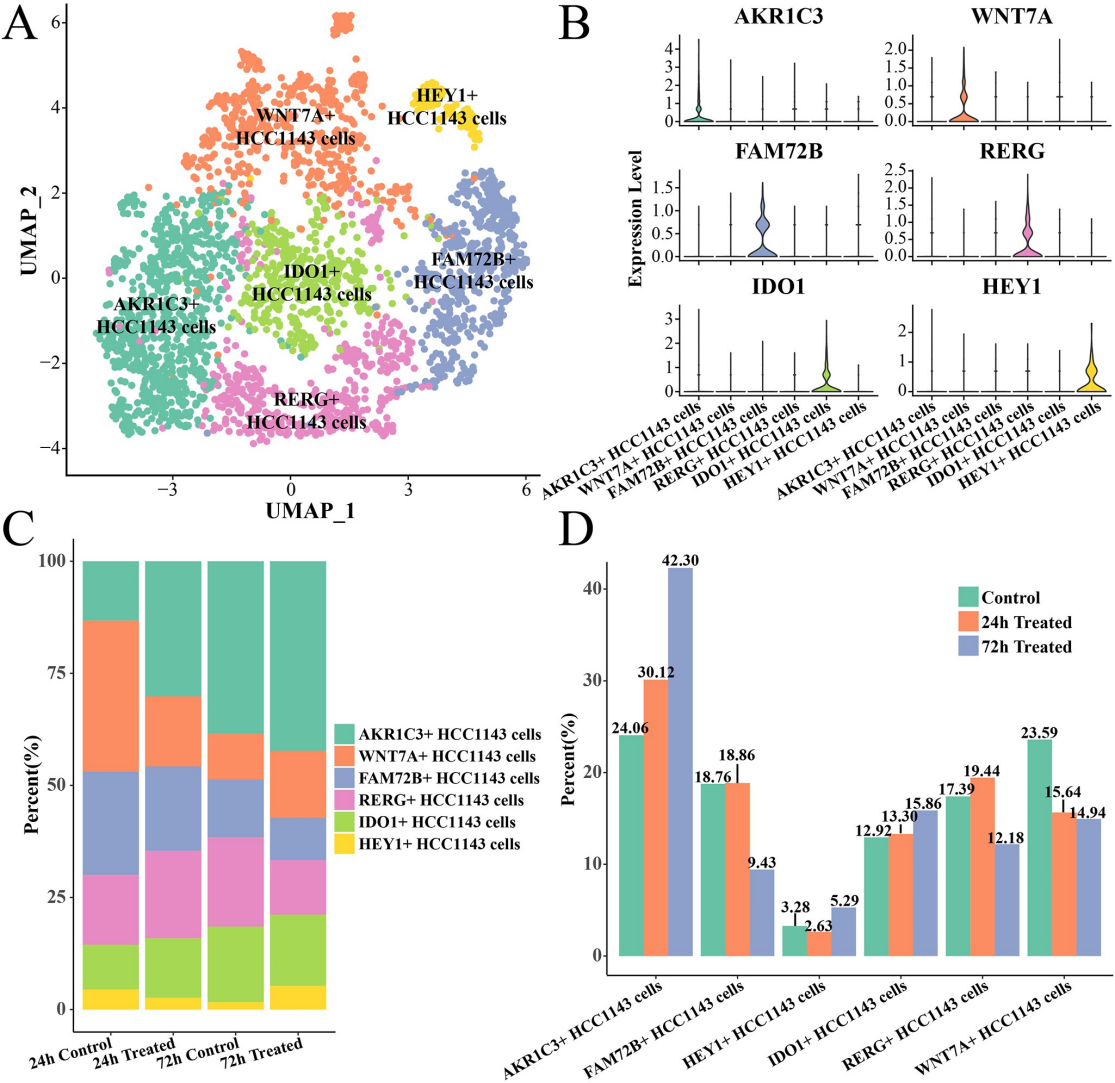
Single cell profile and paclitaxel-resistant subsets analysis. (A) UMAP dimensionality reduction map of HCC1143 cell clusters. (B) Violin plot of marker gene expression level in each cell subset. (C) The proportion of each cell subset in different samples. (D) The proportion of each cell subset in control, 24h treated, and 72h treated groups.

A total of 6 cell subsets (including 2798 cells) were identified after that scRNA-seq data were filtered, normalized, integrated, clustered and annotated (Fig 1A). Based on the highlight expression genes (Fig 1B), they were defined as the AKR1C3+, WNT7A+, FAM72B+, RERG+, IDO1+ and HEY1+ HCC1143 cell subsets. We counted the proportion of each cell subset in control group and experimental group and found that the proportion of AKR1C3+, IDO1+ and HEY1+ cell subsets was higher in 72h treated group than that in 24h treated group (Fig 1C). Specially, the proportion of AKR1C3+HCC1143 cells increased markedly as the extension of paclitaxel treatment time (Fig 1D). Thus, the AKR1C3+, IDO1+ and HEY1+ HCC1143 cells were selected as paclitaxel-resistant subsets, and their dynamic changes of gene expression patterns were further analyzed.

### Pathway difference and function enrichment among paclitaxel-resistant subsets

**Fig 2:**
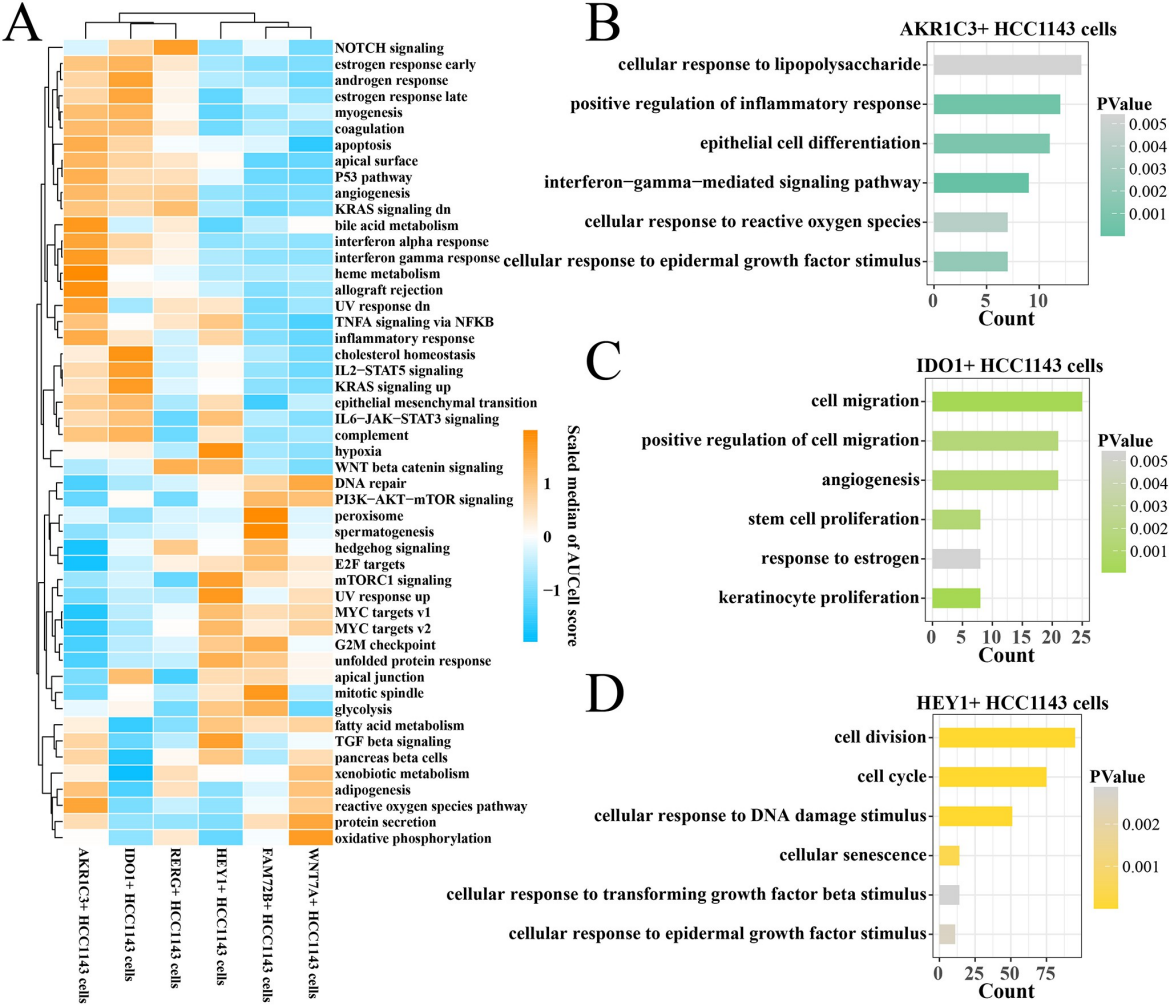
Function enrichment among paclitaxel-resistant subsets. (A) Heatmap of the median hallmark enrichment score for each cell subset. (B) The biological process of highly expressed differential genes in AKR1C3+ HCC1143 cells. (C) The biological process of highly expressed differential genes in IDO1+ HCC1143 cells. (D) The biological process of highly expressed differential genes in HEY1+ HCC1143 cells.

To elucidate the cancer-related pathways enrichment difference of these paclitaxel-resistant subsets, we calculated the hallmark enrichment score (median) of each cell subset and found that the apoptosis, bile acid metabolism, interferon response, heme metabolism and reactive oxygen species (ROS) pathways in AKR1C3+ cell subset was significantly activated (Fig 2A). The hormone response, cholesterol homeostasis, IL2-STAT5 signaling, KRAS signaling up, epithelial mesenchymal transition pathways in IDO1+ cell subset were significantly activated (Fig 2A). The mTORC1 signaling, hypoxia, UV response up and TGF-β signaling pathways in HEY1+ cell subset was significantly activated (Fig 2A). In addition, we found that 692 highly expressed differential genes in AKR1C3+ cell subset were enriched in inflammatory response, interferon-γ signaling and ROS pathways (Fig 2B). 851highly expressed differential genes in the IDO1+ cell subset were enriched in cell migration, angiogenesis, stem cell proliferation, estrogen response pathways (Fig 2C). 1906 highly expressed differential genes in the HEY1+ cell subset were enriched in cell division, cell cycle, DNA damage stimulation, epidermal growth factor stimulation of cellular responses pathway (Fig 2D). The detailed pathway analysis results could be seen in S1 Table.

### Pseudotime analysis of AKR1C3+ HCC1143 cells

**Fig 3:**
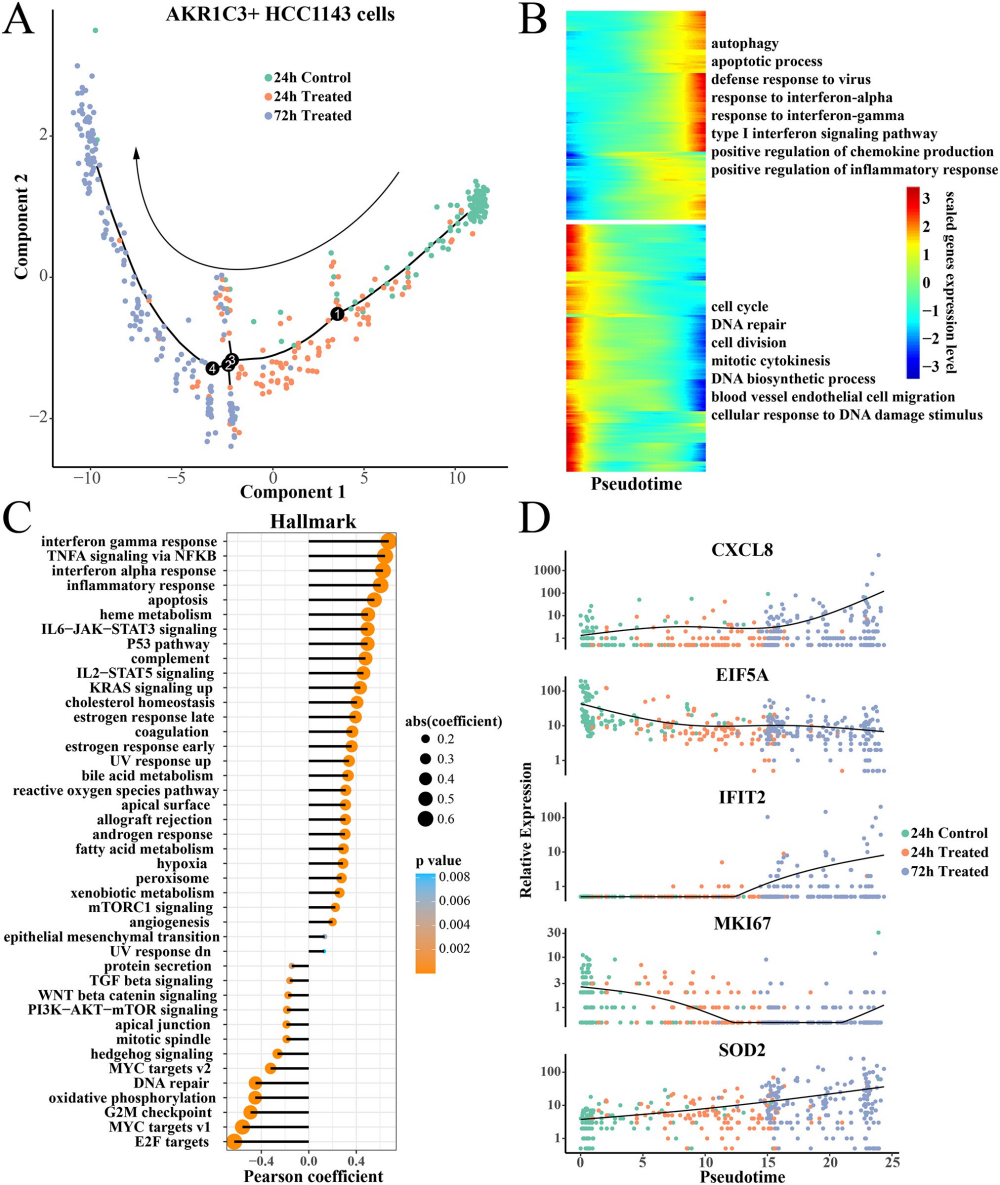
Pseudotime analysis of AKR1C3+ HCC1143 cells. (A) Differentiation trajectory of AKR1C3+HCC1143 cells from 24h control to 24h treatment to 72h treatment. (B) Heatmap of pseudotime related gene expression. (C) The Pearson correlation between the Hallmark score and pseudotime pathway. (D) Scatter plot of Pseudotime-related gene expression at different treatment groups.

In order to further explore the dynamic change of gene expression pattern of AKR1C3+HCC1143 cells with the extension of paclitaxel treatment time, we performed the pseudotime analysis. The control(72h) group was excluded from the data to eliminate the influence of culture time on the expression pattern of tumor cells, and the Control(24h) cells acted as the trajectory start point (Fig 3A). The results showed that the cells treated with paclitaxel (24h) had an obvious trend of differentiation towards 72h paclitaxel treatment (Fig 3A). With the prolongation of pseudotime, the expression levels of defense to virus, response to interferon-alpha, response to interferon-gamma and other inflammatory response-related genes increased gradually (Fig 3B). On the contrary, the expression levels of genes related to cell cycle, DNA repair, DNA biosynthesis, and vascular endothelial cell migration gradually decreased (Fig 3B). In addition, we performed the Pearson correlation analysis between the hallmark enrichment score and pseudotime, and found parallel results. The hallmark score of response to interferon-gamma and other inflammatory response-related pathway displayed a significantly positive correlation with pseudotime (Fig 3C). Significantly different interest genes, such as chemokine CXCL8 [51], interferon induced protein IFIT2 [52], and oxidative stress protein SOD2 expression gradually increased with pseudotime, while the expression of translation factor EIF5A [53] and marker of proliferation Ki-67 (MKI67) [54] showed a downward trend with pseudotime (Fig 3D), this may be correlated with their biological function. The results implied that paclitaxel may activate the inflammatory response and reduce the proliferation ability of AKR1C3+HCC1143 cell lines, which was an interesting phenomenon and needed further validation in other cell subsets.

### Pseudotime analysis of IDO1+HCC1143 cells

**Fig 4:**
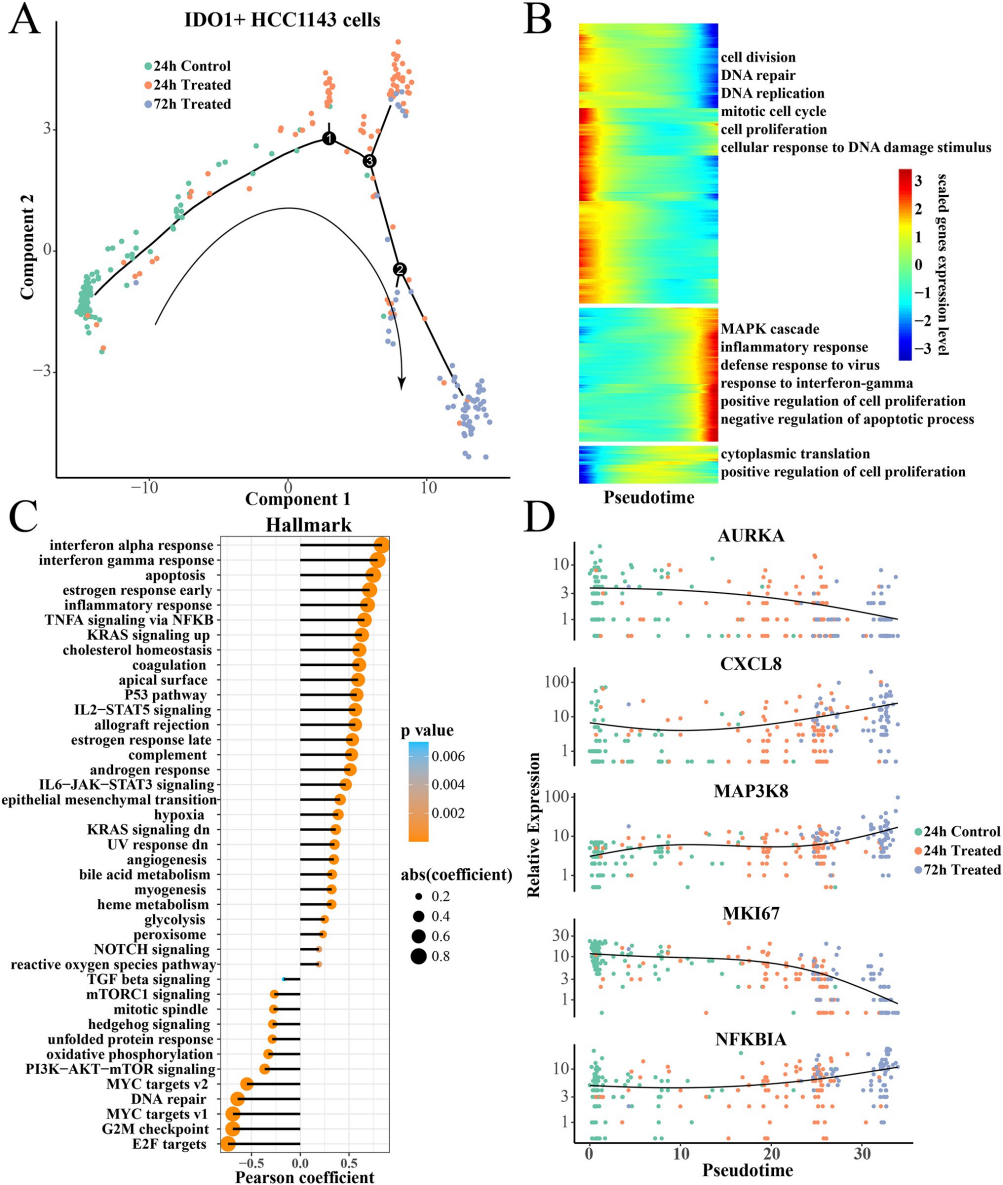
Pseudotime analysis of IDO1+ HCC1143 cells. (A) Differentiation trajectory of IDO1+HCC1143 cells from 24h control to 24h treatment to 72h treatment. (B) Heatmap of pseudotime related gene expression. (C) The Pearson correlation between the Hallmark score and pseudotime pathway. (D) Scatter plot of Pseudotime-related gene expression at different treatment groups.

The same method was used to construct the differentiation trajectory of IDO1+HCC1143 cells (Fig 4A), the control cells had a trend of diverges into two directions (24h and 72h paclitaxel treatment). In particular, with pseudotime prolongation, the expression levels of defense to virus, response to interferon-gamma, inflammatory response to interferon-gamma, inflammatory response and MAPK cascade -related genes increased gradually (Fig 4B), the expression levels of genes related to cell division, DNA repair and DNA replication were gradually decreased (Fig 4B). In the correlation analysis, the hallmark score of interferon, apoptosis, and inflammatory response showed significantly positive correlation with pseudotime (Fig 4C). The key interest genes, such as the chemokine CXCL8, inflammatory regulatory factor NFKBIA and MAP3K8 expression were gradually increased with pseudotime, while the cell cycle-regulated kinase AURKA [55] and MKI67 were gradually decreased with pseudotime (Fig 4D).

### Pseudotime analysis of HEY1+HCC1143 cells

**Fig 5:**
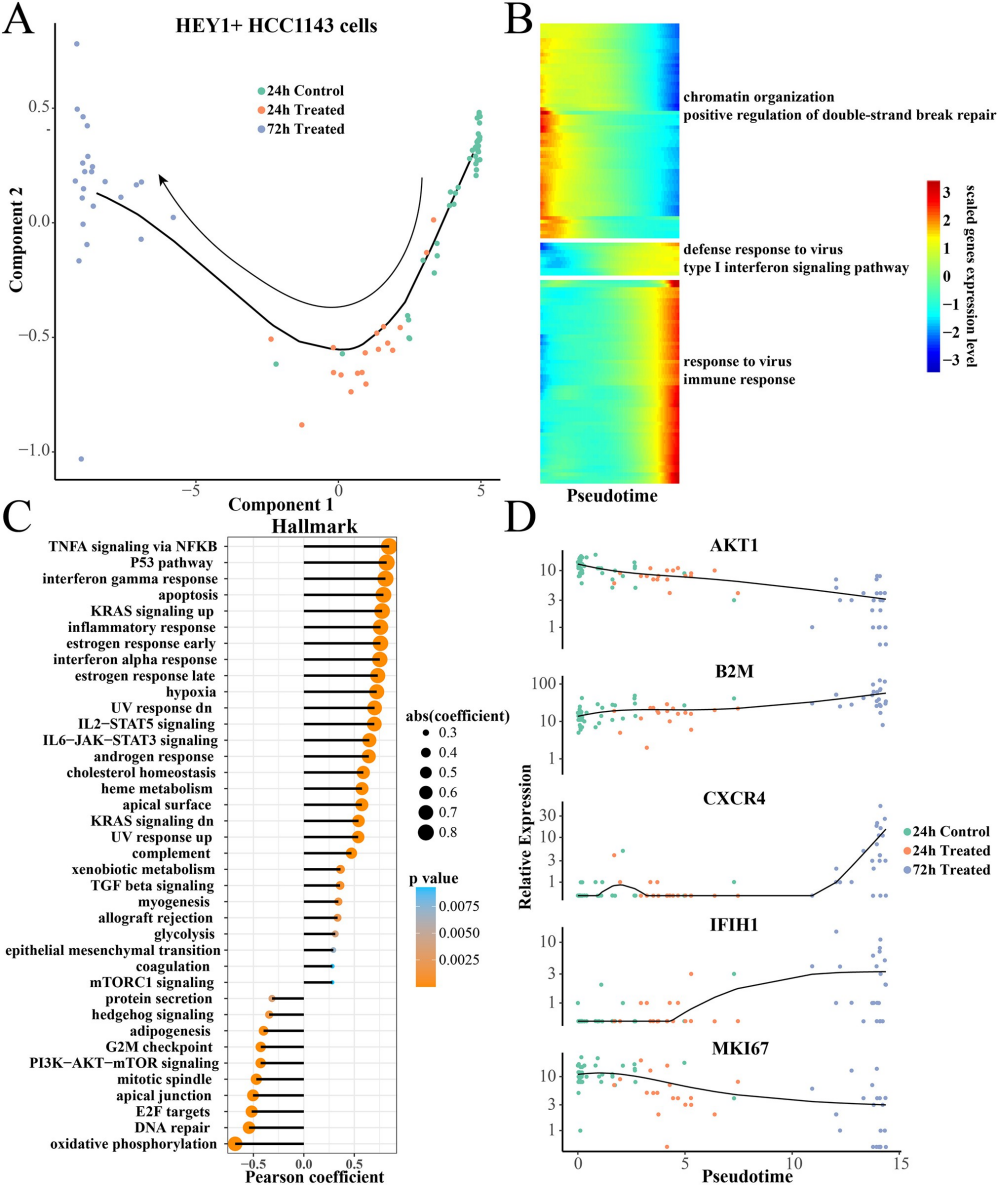
Pseudotime analysis of HEY1+ HCC1143 cells. (A) Differentiation trajectory of HEY1+HCC1143 cells from 24h control to 24h treatment to 72h treatment. (B) Heatmap of pseudotime related gene expression. (C) The Pearson correlation between the Hallmark score and pseudotime pathway. (D) Scatter plot of Pseudotime-related gene expression at different treatment groups.

We constructed the differentiation trajectory of HEY1+HCC1143 cells to explore the dynamic change of gene expression pattern (Fig 5A), the results showed the control cell had an obvious differentiation trend of 72h paclitaxel treatment. Specially, we found that the expression levels of genes enriched in defense to virus, immune response, type I interferon signaling pathway expression levels were gradually raised (Fig 5B). However, the expression level of positive regulation of double-strand break repair related genes in chromatin organization were gradually decreased (Fig 5B). The hallmark score of TNFA signaling via NFKB, P53 pathway, interferon gamma response exhibited distinctly positive correlation with pseudotime (Fig 5C), while the hallmark score of DNA repair, E2F targets, oxidative phosphorylation displayed markedly negative correlation with pseudotime (Fig 5C). The key interest genes, such as histocompatibility complex protein B2M [56], chemokine CXCR4 and interferon induced protein IFIH1 were gradually increased with pseudotime, while the cell cycle-regulated kinase AKT1 [57] and MKI67 expression were gradually decreased with pseudotime (Fig 5D)

### Identifying of transcription factor among paclitaxel-resistant subsets

**Fig 6:**
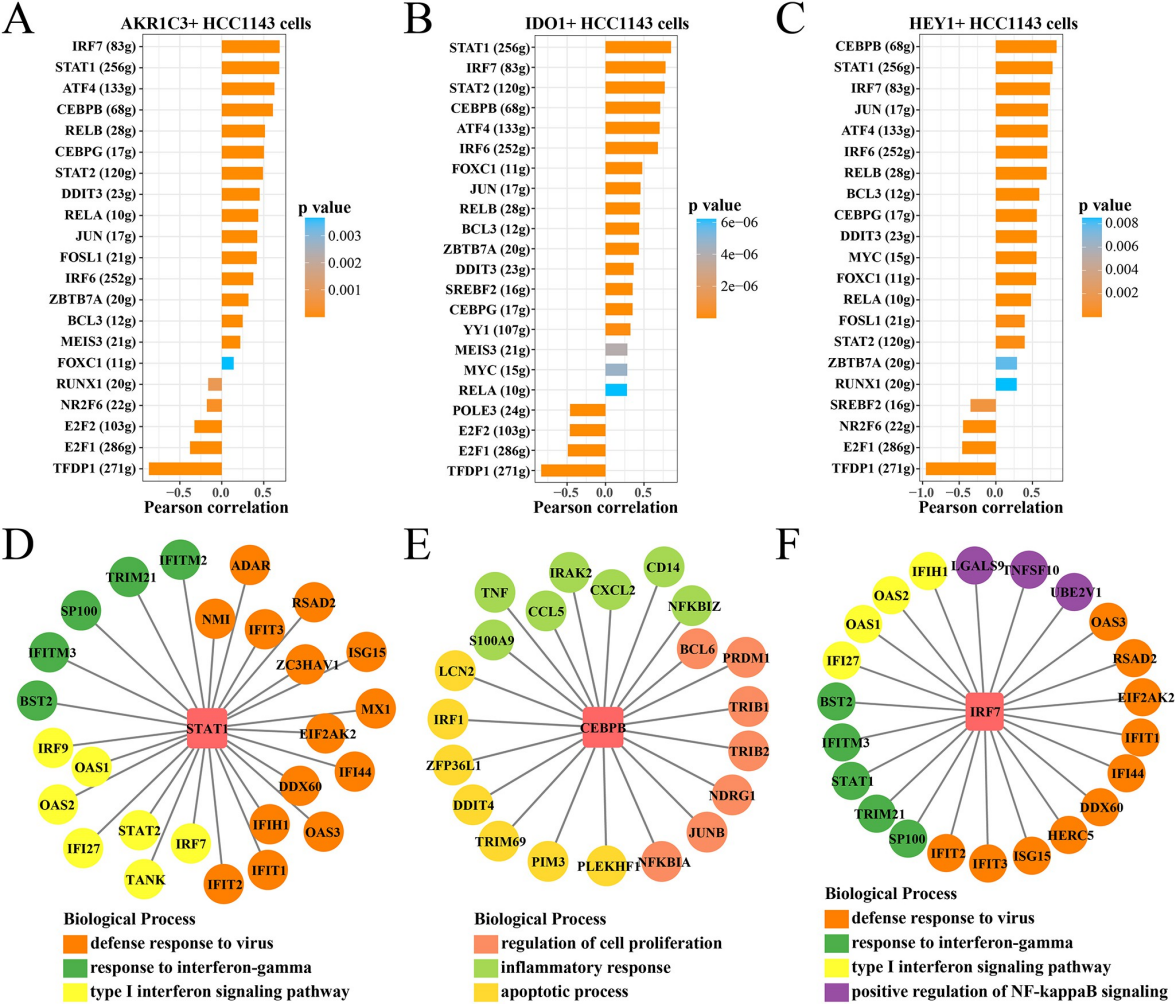
Identifying of transcription factor among paclitaxel-resistant subsets. (A) The Pearson correlation between regulon score and pseudotime pathway in the AKR1C3+HCC1143 cells. (B) The Pearson correlation between regulon score and pseudotime pathway in the IDO1+HCC1143 cells. (C) The Pearson correlation between regulon score and pseudotime pathway in the HEY1+HCC1143 cells. (D) The regulatory network of STAT1. (E) The regulatory network of CEBPB. (F) The regulatory network of IRF7.

To identify the paclitaxel-resistant TFs, we conducted the SCENIC analysis to find the potential regulons and calculated the AUCell activity score of each regulon in the AKR1C3+HCC1143 cells, IDO1+HCC1143 cells and HEY1+HCC1143 cells. Then, we analyzed the Pearson correlation between the regulon and pseudotime and found that TFs such as STAT1, IRF7, CEBPB, STAT2 and ATF4 in these cell subsets were significantly positively correlated (Fig 6A–6C). We focused on the top 2 TFs with the highest correlation in each cell subset, the results showed that STAT1 and its target genes involved in the defense response to virus, response to interferon-gamma and type I interferon signaling process (Fig 6D). CEBPB and its target genes involved in regulation of cell proliferation, inflammatory response and apoptotic process (Fig 6E). IRF7 regulation network was involved in defense response to virus, response to interferon-gamma, type I interferon and positive regulation of NF-kappaB signaling process (Fig 6F).

### Identifying inhibitor of paclitaxel resistance-related TFs

**Fig 7:**
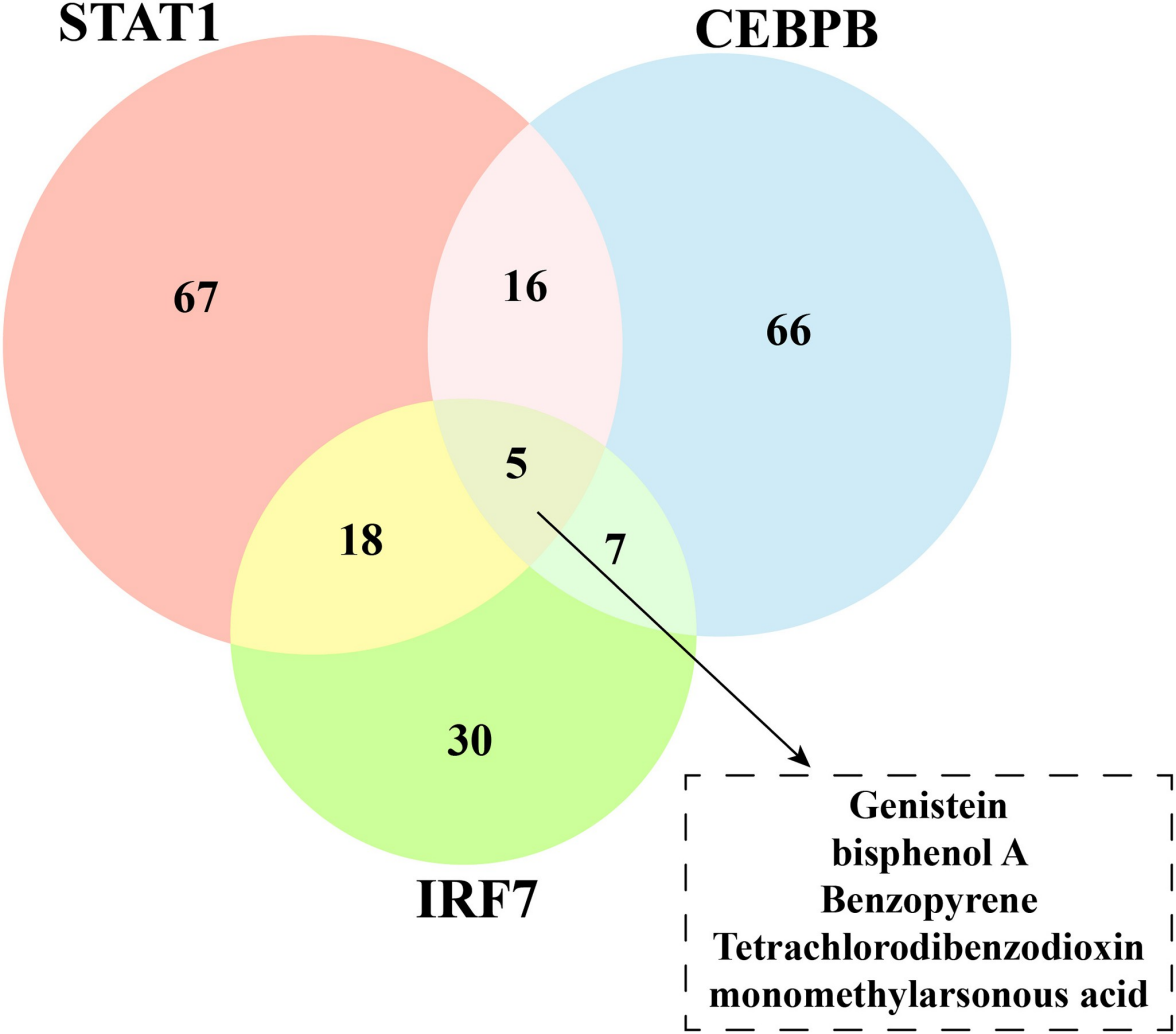
Identifying of transcription factor inhibitors. Venn plot of transcription factor inhibitors.

Finally, we uploaded these genes STAT1, CEBPB and IRF7 to the CTD database, and found five drugs: Genistein, bisphenol A, Benzopyrene, Tetrachlorodibenzodioxin and monomethylarsonous acid as inhibitors targeting these TFs (Fig 7). These five drugs may further expand the strategy of combining paclitaxel with other drugs to treat triple-negative breast cancer.

## Discussion

TNBC is the most malignant subtype of breast cancer with high degree of aggressiveness [58]. In this study, we conducted a comprehensive analysis of scRNA-seq data of TNBC cells treated with paclitaxel at different points (24 and 72h) to explore the potential resistance mechanisms of TNBC cells to paclitaxel. Firstly, 6 cell subsets AKR1C3+, WNT7A+, FAM72B+, RERG+, IDO1+ and HEY1+HCC1143 cells were annotated, among which AKR1C3+, IDO1+ and HEY1+ cells were regarded as paclitaxel resistance subsets. Finally, we identified STAT1, CEBPB and IRF7 as key TFs for paclitaxel resistance in TNBC therapy. And their inhibitors such as bisphenol A and Benzopyrene could be used as combination therapies of paclitaxel to improve the survival outcomes of patients with TNBC.

In three paclitaxel resistance subsets, their marker genes conferred the cells to paclitaxel resistance through different signaling pathways. AKR1C3 is known as an aldo-keto reductase [59], which could be used the Nicotinamide Adenine Dinucleotide Phosphate (NADP) as a coenzyme to reduce anthracyclines to hydroxyl metabolites, causing tumors to develop resistance to carbonyl-containing drugs such as doxorubicin and daunorubicin [60]. The anthracycline induced the tumor apoptosis through reactive oxygen species (ROS) activation, while the malignant cells become drug-resistant by upregulating the AKR1C3 to reduce ROS activity [61]. In addition, the overexpression of AKR1C3 may lead to the loss of tumor suppressor genes PTEN and activate the PI3K/AKT pathway, which eventually induces cell proliferation and avoids apoptosis [62]. Interestingly, these functions were also well predicted in AKR1C3+ HCC1143 cell pathway analysis. IDO1 is a tryptophan metabolizing enzyme and mediates the tryptophan depletion to escape cytotoxic T cell killing, as a core element in tumor-promoting inflammation [63], the IDO inhibitor therapy have confirmed that IDO1 reduces the role of tumor immune response [64]. Additionally, newly published papers revealed that IDO1 also could facilitate tumor neovascularization by disturbing local innate immunity [65]. HEY1 as a transcriptional suppressor associated with cell division is involved in the epithelial-mesenchymal transition (EMT), which benefits the migration, invasion and anti-apoptosis of tumor cells [66]. Moreover, the HEY1 as the signaling downstream target of Notch also activates the PI3K/AKT pathway to promote migration and invasion [67]. Based on above studies, these cell subgroups may play different role in paclitaxel resistance. The AKR1C3+ cells could catalyze the paclitaxel to the non-toxic hydroxyl metabolites, accompanied with activated ROS and inflammation; the IDO1+ cells may inhibit T cell function by consuming tyrosine and HEY1+ cells enhanced the cell proliferation to promote tumor survival. Collectively, they contributed to paclitaxel resistance in tumor cells through complex signaling rewiring and interaction.

We calculated the hallmark score of these paclitaxel resistance subsets, the AKR1C3+ and HEY1+ cell subsets had the similar enrichment pathway, in which the hormone, interferon and inflammatory response, apoptosis, P53 pathway and the fatty acid metabolism pathway were significantly activated. As we all known that inflammation is a host’s protective response to infection and tissue damage, however, the inflammation is also an important hallmark of cancer progression. In the context of cancer, in contrast to wound healing where immune cell recruitment and epithelial cell proliferation subside, DNA is destroyed by the tumor microenvironment and the growing tumor is in a state of constant stress [68]. The stress response promoted the activation of bypass and alternative signaling pathways [69], leading to the pro-tumor signaling pathways TNFA signaling via NFKB, IL6-JAK-STAT3 signaling and IL2-STAT5 signaling were strongly activated, this mechanism of promoting paclitaxel resistance also appears in vitro.

Common drug resistance refers to the initial sensitivity of tumor cells to chemotherapy drugs. After a period of time, due to chemotherapy drugs inducing the body to produce some resistance genes, tumor cells evolving, or gene mutations occurrence, leading to resistance to chemotherapy drugs [70]. Researches have disclosed that the progression of TNBC chemoresistance is complex, involving in interactions among tumor microenvironment, drug efflux, tumor stem cells [71], and bulk tumor cells, which are manipulated by kinds of signals. Furthermore, TNBC’s high heterogeneity is a major brake to successful therapy [72, 73]. Through scRNA-seq analysis, we identified three paclitaxel-resistant IFs STAT1, CEBPB, and IRF7. Signal transducer and activator of transcription 1 (STAT1) is a cancer associated gene, which is involved in the cytokines (interferon-α/γ and interleukin-6) and growth factor response [74]. When external stimulation signals were transmitted into the cytoplasm, the STAT1 is phosphorylated and dimerized, and as an activating transcription factor, it entered the nucleus to enhance targeted genes transcription. Some studies reported that activation of STAT1 could promote the apoptosis of tumor cells [75], while another studies reported that high levels of ROS promote JAK2 phosphorylation, leading to the pJAK2-STAT1 signaling mediated anti-apoptosis of tumor cells [76], this may explain why high levels of inflammation promote drug resistance in tumor cells. Additionally, Fludarabine, a well-known STAT1 inhibitor has been adopted for clinical lymphodepleting chemotherapy asiatance [77, 78]. CEBPB is an important cytokines response transcription factor, the chemokines CXCL12 induced recruitment of Tregs via CEBPB/NF-κB signaling to promote drug resistance [79], thus the inhibition of CEBPB benefited the tumor treatment. Homoharringtonine, a kind of alkaloid, could be regarded as the inhibitor of CEBPA, because it could repress the synthesis of CEBPA and decrease CEBPA protein levels, which may possibly be the mechanism of Homoharringtonine in CEBPA-double-mutant acute myeloid leukemia [80]. Interferon regulator factor 7 (IRF7) stimulates the transcription of interferon genes, which ultimately triggers an interferon response [81], while the high production of interferon will increase the degree of inflammatory response and promote paclitaxel resistance in TNBC. MyD88 [82] and TARBP2 [83] are two newly found inhibitors for IRF7, which are worth further research in the future. Collectively, these three genes could be the resistance genes for paclitaxel in TNBC treatment, and their mutation need deep research in the future. The limitations of this study lies in lack of web experiment to validate the functions of paclitaxel resistance related TFs. In the future, we plan to preliminarily conduct cell experiment to detect the expression levels of these TFs in TNBC cells. Then we silence highly expressed TFs, and treat cells with different concentrations of paclitaxel to assess cell cytotoxicity by calculating half maximal inhibitory concentration. Overall, these three key TFs could be the potential therapeutic targets, and their practical role will be further confirmed in clinical treatment.

## Conclusion

Collectively, three paclitaxel resistance relevantTFs STAT1, CEBPB and IRF7 were identified, and they shared 5 common inhibitors (Genistein, bisphenol A, Benzopyrene, Tetrachlorodibenzodioxin and monomethylarsonous acid). Our study provides fundamental molecular clues for the mechanism of paclitaxel resistance and helpful instrument for the combination of paclitaxel therapy to improve chemotherapy efficacy of TNBC patients.

## Supporting information

S1 Table Detailed enrichment analysis results. (XLSX)

## Abbreviations

cds: CellDataSet
CML: Chronic Myeloid Leukemia
CTD: Comparative Toxicogenomics Database
DAVID: Database for Annotation, Visualization and Integrated Discovery
DEGs: Differentially expressed genes
DMSO: dimethyl sulfoxide
EGFR: Epidermal Growth Factor Receptor
ER: estrogen receptors
ERK: extracellular regulated protein kinases
GEO: Gene Expression Omnibus
GRNs: gene regulatory networks
HER2: human epidermal growth factor receptor-2
MAPK: mitogen activated protein kinases
MSigDB: Molecular Signatures Database
PCA: principal component analysis
PCR: pathologic complete response
PR: progesterone receptors
RTKs: receptor tyrosine kinases
SCENIC: Single-cell regulatory network inference and clustering
scRNA-seq: single-cell RNA-sequencing
TNBC: triple negative breast cancer
UMAP: Uniform Manifold Approximation and Projection for Dimension Reduction
